# Correction: Evaluation of community based surveillance in the Rohingya refugee camps in Cox’s Bazar, Bangladesh, 2019

**DOI:** 10.1371/journal.pone.0299167

**Published:** 2024-02-14

**Authors:** Elburg Van Boetzelaer, Samiur Chowdhury, Berhe Etsay, Abu Faruque, Annick Lenglet, Anna Kuehne, Isidro Carrion-Martin, Patrick Keating, Martins Dada, Jorieke Vyncke, Donald Sonne Kazungu, Maria Verdecchia

There are errors in the Funding and Competing Interests statements. The correct statements are:

**Funding:** Medecins sans Frontières (MSF) provided support in the form of salaries for EVB, SC, BT, AL, AK, ICM, PK, MV, MD, JV, and DSK. The specific roles of these authors are articulated in the ‘author contributions’ section. MSF programmatic funding covered all other costs associated with the study. The funder otherwise had no role in study design, data collection and analysis, decision to publish, or preparation of the manuscript.

**Competing Interests:** The authors have read the journal’s policy and have the following competing interests to declare: Medecins sans Frontières (MSF) provided support in the form of salaries for EVB, SC, BT, AL, AK, ICM, PK, MV, MD, JV, and DSK. This does not alter our adherence to *PLOS ONE* policies on sharing data and materials. There are no patents, products in development, or marketed products associated with this research to declare.
