# Supplementary figures and images for: Evaluation of community based surveillance in the Rohingya refugee camps in Cox’s Bazar, Bangladesh, 2019
Source: PLoS One. 2020 Dec 23;15(12):e0244214. doi: 10.1371/journal.pone.0244214 (PMC7757896; doi:10.1371/journal.pone.0244214)

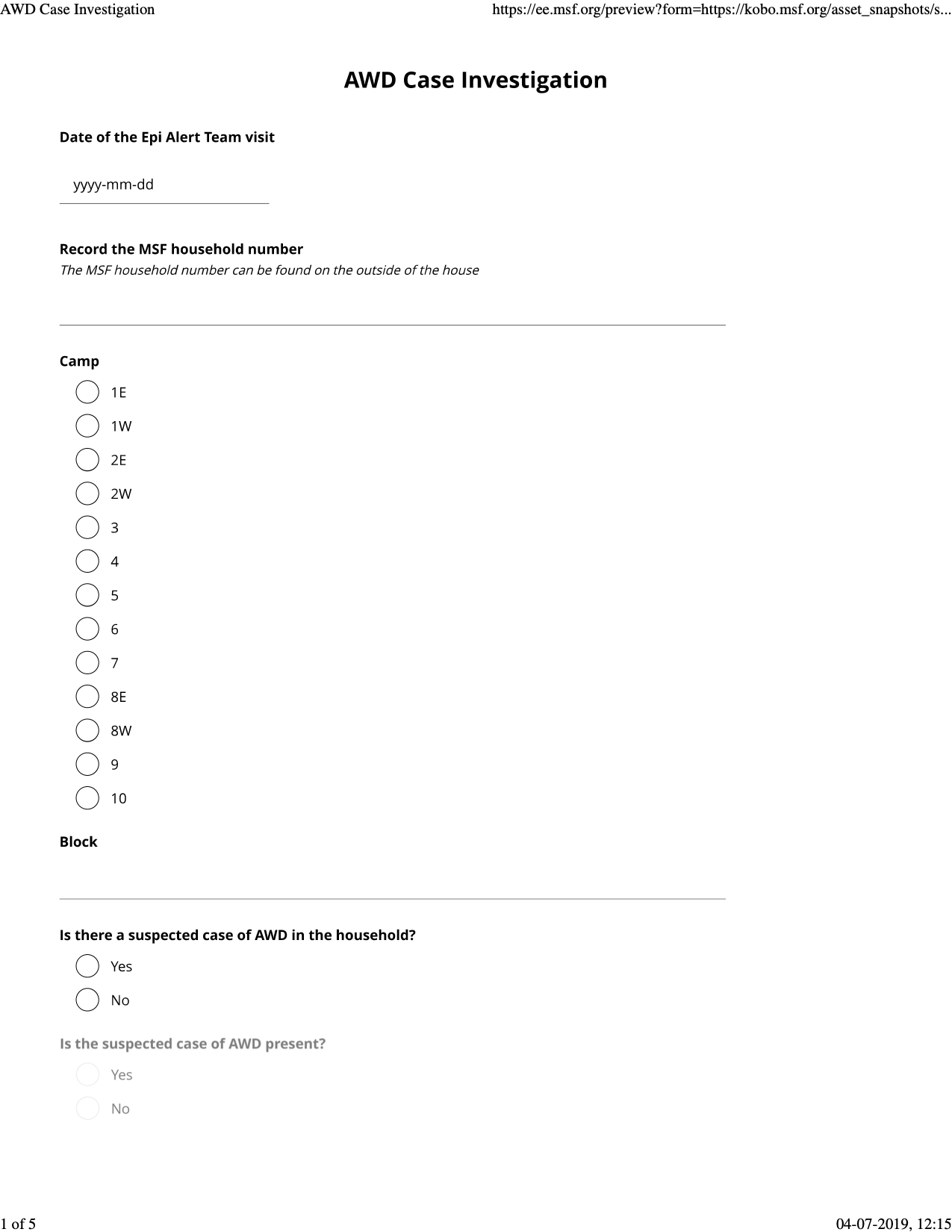

Supplement: S1 File — (TIFF) [file pone.0244214.s001.tiff]
