# Supplementary material for: Evaluation of community based surveillance in the Rohingya refugee camps in Cox’s Bazar, Bangladesh, 2019
Source: PLoS One. 2020 Dec 23;15(12):e0244214. doi: 10.1371/journal.pone.0244214 (PMC7757896; doi:10.1371/journal.pone.0244214)
Supplement: Attachment — Submitted filename: review2.pdf [file pone.0244214.s002.pdf]

Dear Authors,

I think the paper is much clearer now, thank you for your commitment. I believe this paper should be published, this is an important experience which involved a lot of resources including hundreds of committed colleagues during an extended period of time.

I just have two major issues that I think should be taken into consideration before publication and few minor comments.

Major issues:

- **Data quality / validity evaluation attribute**:
  - In Table 3, the measurement modality for the "*Data quality / validity evaluation attribute*" is stated as "*Comparison of trends of health facility surveillance (gold standards) and community based surveillance trends*".
  - The fact is that **you can't compare these trends as they don't have the same population under surveillance**.
    - You correctly say it in the discussion lines 357-360: "*While the trends of reported suspected cases from all the components of the surveillance system were similar, and could be an indicator of data quality and validity, **it must be noted that the catchment population of the CBS and health facility surveillance differed** as the health facilities were also visited by patients who did not live in the CBS catchment area.*"
  - In any case, **it is misleading to evaluate this "*Data quality / validity*" attribute, as you have no data to do it.**
    - Lines 240-249: when you compare the "*Surveillance worker curve*" and the "*Epi alert team curve*", you are not evaluating the "*data quality/validity*" but the evolution of the PPV. The assertion "*The trend comparison per disease show that surveillance workers identified the most suspected cases*" makes no sense here, due to your design, indeed the surveillance workers will always detect more suspect cases that the Epi or Response teams (who are coming after a case has been detected by the surveillance worker).
  - Please consider the following actions:

* Abstract line 25, methods line 215, Table 3, results lines 240-249: **remove the data quality/validity attribute** as it cannot be evaluated (see above).
* **From lines 241-249, you can move the following slightly rephrased sentence and figures 4 to 7 to the PPV results section**: "*Available health facility surveillance data from two MSF health facilities and data from the CBS (suspected cases identified by the surveillance workers and verified by the Epi Alert Team) are shown for AWD, AJS, Diphtheria and measles (Figure 4-7).*"

- **CBS case definitions**:
  - **CBS workers mostly used the exact same case definitions for suspect cases that at the health facility**, as correctly mentioned lines 182-184: "*The same diseases that were included in the CBS were under surveillance at the health facilities using the same MSF case definitions (Table 1).*"
  - **These were not "*community case definitions*" as the term is generally understood.** For example, the following ones are community case definitions: acute jaundice: "*Any person with a sudden yellowing of the skin for not more than two weeks, with or without elevated body temperature.*"; Acute flaccid paralysis: "*Any child with a sudden onset of acute paralytic disease.*" (2014 WHO guide: https://www.afro.who.int/sites/default/files/2017-06/a-guide-for-establishing-community-based-surveillance-102014_0.pdf). Other examples of guides providing such "*community case definitions*": Integrated Diseases Surveillance and Response in the African Region. Community-based Surveillance (CBS) Training Manual. WHO, 2015.; Community-based surveillance: guiding principles. IFRC, 2017.; Technical Guidelines for Integrated Disease Surveillance and Response in the African Region; WHO and US CDC, 2010.
  - To avoid any misunderstanding, please consider the following actions:
    * Line 101: **Replace** "*The CBS system used community case definitions (Table 1)*" by "*The CBS system used standard case definitions (Table 1)*".
    * Table 1: **Replace column titles**: "*CBS case definition (suspected)*" by

“*Case definitions used by CBS surveillance workers*” and “*Health facility case definition*” by “*Case definitions used at health facility*”.

  - You may, or not, discuss around lines 347-353 how using “*community case definitions*” could have improved the ability of community volunteers to detect true disease.

Minor issues:

- Abstract line 35 and Discussion line 335:
  - “*The CBS allowed for timely detection and response […]*”. Indeed timely response is correct, but for timely detection this doesn’t seem correct: visits were only on a monthly basis and you don’t have data on the delay between onset of symptoms and detection.
  - Consider rephrasing the sentence to “*The CBS allowed for timely response […]*”.
- Figure 1:
  - You mention in your reply that “*We have moved the figure with the overview of the surveillance system to the end of the ‘methods’ section, line 204, as suggested*”. But, you have not, it is still referred line 73 and its mention doesn’t appear later on.
  - To clarify that surveillance workers referred to the health facilities and that Epi and Response team reminded visited patients to go to the health facility, consider modifying in the figure: “*Referral by Epi Alert Team […]*” by “*Epi Alert Team or Medical Response Team reminded suspected cases to visit the nearest health facility*”.
  - “*Weekly upload of case information suspected case on EWARS*” is unclear, consider “*Weekly upload of case information on EWARS*” OR “*Weekly upload of information on suspected cases on EWARS*”.
- Line 58: the wording “Some CBS train community volunteers to use actively search for cases of pre-defined case definition […]” is unclear, consider the following re-

wording: "*Some CBS train community volunteers to actively search for cases using pre-defined case definitions […].*"

- In your reply you mention: "*We have added that the households were visited by 'a surveillance worker' (single)*". But, you have not, only "*surveillance workers*" (plural) is mentioned in the 4 occurrences of "*surveillance workers*" visiting households. Consider correcting these four occurrences.
- The number of surveillance workers per team leader is currently mentioned in the results section (line 306), consider moving it to the Methods section, potentially in table 2.
- Figures 4 to 7: you need to add different line types (or use different colours) so that it is easy to understand which line represents what.
- Line 396: don't use the acronym HR, but the full wording: "*human resources*" (if this is what HR meant).
