# Supplementary material for: Evaluation of community based surveillance in the Rohingya refugee camps in Cox’s Bazar, Bangladesh, 2019
Source: PLoS One. 2020 Dec 23;15(12):e0244214. doi: 10.1371/journal.pone.0244214 (PMC7757896; doi:10.1371/journal.pone.0244214)
Supplement: Attachment — Submitted filename: Response to Reviewers 2.docx [file pone.0244214.s003.docx]

Oslo, 30 November 2020

Dear Editor,

We would like to thank you for the consideration of our manuscript ‘Description of Community Based Surveillance in the Rohingya refugee camps in Cox’s Bazar, Bangladesh, 2019’ for publication in PlosOne. In addition, we would like to thank both reviewers very much for taking the time to review our draft manuscript thoroughly and share such insightful comments. We have addressed all comments, kindly find our responses below.

Kind regards, on behalf of the authors,

Elburg van Boetzelaer (corresponding author)

Reviewer #1

**Major issues:**

- **Data quality / validity evaluation attribute**:
  - In Table 3, the measurement modality for the “*Data quality / validity evaluation attribute*” is stated as “*Comparison of trends of health facility surveillance (gold standards) and community based surveillance trends*”.
  - The fact is that **you can’t compare these trends as they don’t have the same population under surveillance**.

* You correctly say it in the discussion lines 357-360: “*While the trends of reported suspected cases from all the components of the surveillance system were similar, and could be an indicator of data quality and validity,* ***it must be noted that the catchment population of the CBS and health*** ***facility surveillance differed*** *as the health facilities were also visited by*

*patients who did not live in the CBS catchment area.*”

**–** In any case, **it is misleading to evaluate this “*Data quality / validity*” attribute, as you have no data to do it.**

* Lines 240-249: when you compare the “*Surveillance worker curve*” and

the“*Epi alert teamcurve*”, you are not evaluating the“*data quality/validity*” but the evolution of the PPV. The assertion “*The trend comparison per diseases how that surveillance workers identified the most suspected cases*” makes no sense here, due to your design, indeed the surveillance workers will always detect more suspect cases that the Epi or Response teams (who are coming after a case has been detected by the surveillance worker).

**–** Please consider the following actions:

- Abstract line 25, methods line 215, Table 3, results lines 240-249: **remove the data quality/validity attribute** as it cannot be evaluated (see above). We have made the suggested edits.
- **From lines 241-249, you can move the following slightly rephrased sentence and figures 4 to 7 to the PPV results section**: “*Available health facility surveillance data from two MSF health facilities and data from the CBS (suspected cases identified by the surveillance workers and verified by the Epi Alert Team) are shown for AWD, AJS, Diphtheria and measles* *(Figure 4-7).*” We have made the suggested edits.
- **CBS case definitions**:
  - **CBS workers mostly used the exact same case definitions for suspect cases that at the health facility**, as correctly mentioned lines 182-184: “*The same diseases that were included in the CBS were under surveillance at the health facilities using the same MSF case definitions (Table 1).*”
  - **These were not “*community case definitions*” as the term is generally understood.** For example, the following ones are community case definitions: acute jaundice: “*Any person with a sudden yellowing of the skin for not more than two weeks, with or without elevated body temperature.*”; Acute flaccid paralysis: “*Any child with a sudden onset of acute paralytic disease.*” (2014

WHOguide: https://www.afro.who.int/sites/default/files/2017-06/a-guide-forestablishing-community-based-surveillance-102014_0.pdf ). Other examples of guides providing such “*community case definitions*”: Integrated Diseases Surveillance and Response in the African Region. Community-based Surveillance (CBS) Training Manual. WHO, 2015.; Community-based surveillance: guiding principles. IFRC, 2017.; Technical Guidelines for Integrated Disease Surveillance and Response in the African Region; WHO and US CDC, 2010. **–** To avoid any misunderstanding, please consider the following actions:

* Line 101: **Replace** “*The CBS system used community case definitions (Table 1)*” by “*The CBS system used standard case definitions (Table 1)*”. We have made the suggested edit.

* Table 1: **Replace column titles**: “*CBS case definition (suspected)*” by “*Case definitions used by CBS surveillance workers*” and “*Health facility*

*case definition*” by “*Case definitions used at health facility*”. We have made the suggested edits.

- You may, or not, discuss around lines 347-353 how using “*community case*

*definitions*” could have improved the ability of community volunteers to detect true disease. Thank you for this suggestion. However, we have decided not to include this in the discussion as it did not become apparent from this evaluation that the case definitions were an obstacle to community based surveillance workers detecting true disease.

**Minor issues**:

- Abstract line 35 and Discussion line 335:
  - “*The CBS allowed for timely detection and response […]*”. Indeed timely

response is correct, but for timely detection this doesn’t seem correct: visits

were only on a monthly basis and you don’t have data on the delay between onset of symptoms and detection.

- Consider rephrasing the sentence to “*The CBS allowed for timely response […]*”. Thank you for pointing this out. We have made the edits.
- Figure 1:
  - You mention in your reply that “*We have moved the figure with the overview of the surveillance system to the end of the ‘methods’ section, line 204, as suggested*”. But, you have not, it is still referred line 73 and its mention doesn’t appear later on. Apologies for this oversight, we have now moved the figure to the end of the methods section.
  - To clarify that surveillance workers referred to the health facilities and that Epi and Response team reminded visited patients to go to the health facility, consider modifying in the figure: “*Referral by Epi Alert Team […]*” by “*Epi Alert Team or Medical Response Team reminded suspected cases to visit the*

*nearest health facility*”. We made the suggested edit.

- “*Weekly upload of case information suspected case on EWARS*” is unclear, consider “*Weekly upload of case information on EWARS*” OR “*Weekly upload*

*of information on suspected cases on EWARS*”. We made the suggested edit.

- Line 58: the wording “Some CBS train community volunteers to use actively search for cases of pre-defined case definition […]” is unclear, consider the following rewording: “*Some CBS train community volunteers to actively search for cases using pre-defined case definitions […].*” We made the suggested edit.
- In your reply you mention: “*We have added that the households were visited by ‘a surveillance worker’ (single)*”. But, you have not, only “*surveillance workers*” (plural) is mentioned in the 4 occurrences of “*surveillance workers*” visiting households. Consider correcting these four occurrences. We believe we have made the suggested edits (line 106-138).
- The number of surveillance workers per team leader is currently mentioned in the results section (line 306), consider moving it to the Methods section, potentially in table 2. We have added the number of surveillance workers per team leader to Table 2.
- Figures 4 to 7: you need to add different line types (or use different colours) so that it is easy to understand which line represents what. We have made the edits as suggested.
- Line 396: don’t use the acronym HR, but the full wording: “*human resources*” (if this is what HR meant). We have made the suggested edit.
